# Different effects of propofol and dexmedetomidine on preload dependency in endotoxemic shock with norepinephrine infusion: a randomized case-control study

**DOI:** 10.1186/cc13607

**Published:** 2014-03-17

**Authors:** T Yu, C Pan, S Liu, F Guo, F Longhini, Y Yang, H Qiu

**Affiliations:** 1 Zhong-Da Hospital, Southeast University, Nanjing, China; 2 Eastern Piedmont University ‘A. Avogadro, Novara, Italy

## Introduction

Septic shock is a status characterized by the simultaneous use of vasopressors and sedative drugs in critical care patients. Little is known about the possible interference of these drugs during shock status. We aimed to clarify how propofol could differently affect the preload dependency in an endotoxemic model in comparison with dexmedetomidine, by evaluation of the systemic vascular system and cardiac function.

## Methods

After animal preparation and under PiCCO monitoring (BL), endotoxemic shock was induced by an intravenous bolus of lipopolysaccharide (LPS, 055:B5) in ketamine-anesthetized rabbits. After fluid resuscitation and norepinephrine infusion (SD0), animals were randomized to propofol (PROP, *n *= 8) or dexmedetomidine (DEX, *n *= 8) sedation, with two incremental doses (SD1 and SD2). The mean arterial pressure (MAP) and central vein pressure (CVP) were monitored. Pulse pressure variation (PPV) and stroke volume variation (SVV) were assessed to evaluate the preload dependency. Global end-diastolic volume, vascular resistances, mean systemic filling pressure, cardiac functional index and vascular resistances were assessed at each time point. Normality was assessed by the Kolmogorov-Smirnov test. Twoway analysis of variance for repeated measures was applied, and the Student-Newmann-Keuls *post-hoc *test when indicated. *P *< 0.05 was considered significant.

## Results

At the increasing dose of propofol, both PPV and SVV significantly increased in SD1 versus SD0 (*P *< 0.01) and SD2 versus SD0 (*P *< 0.001), but only PPV in SD2 versus SD1 (*P *= 0.024). Dexmedetomidine infusion did not affect PPV and SVV. At SD1 and SD2, PPV and SVV were higher in PROP with respect to DEX (*P *< 0.001). Moreover, propofol infusion increased the heart rate and had no effects on cardiac contractility and vascular resistances. On the contrary, in the DEX group we recorded a significant decrease in heart contractility and increment of vascular resistances at SD2. Nonetheless, both drugs affected the MAP, CVP, mean systemic filling pressure, global end-diastolic volume and venous return.

## Conclusion

In an endotoxemic shock model, after fluid resuscitation and during norepinephrine infusion, propofol increased more PPV and SVV in comparison with dexmedetomidine. At high dosage the vascular resistances and heart contractility were influenced by dexmedetomidine, but not by propofol.

